# Global Intensity-Duration-Frequency curves based on observed sub-daily rainfall (GSDR-IDF)

**DOI:** 10.1038/s41597-026-06858-4

**Published:** 2026-02-14

**Authors:** Amy C. Green, Selma B. Guerreiro, Hayley J. Fowler

**Affiliations:** School of Engineering and Tyndall Centre for Climate Change Research, Newcastle University, Newcastle upon Tyne, UK

**Keywords:** Hydrology, Climate change, Hydrology

## Abstract

Short-duration extreme rainfall events can cause flash flooding and infrastructure failures, yet resources to assess these remain limited, particularly at the global scale. Heterogeneous data availability, inconsistent quality control, and methodological differences hinder the development of comparable intensity-duration-frequency (IDF) estimates. To address this gap, we present GSDR-IDF, a global dataset of intensity-duration-frequency curves derived from the largest quality-controlled sub-daily rain gauge dataset: the Global Sub-Daily Rainfall dataset (GSDR), comprising +24,000 hourly rain gauge records for all major climate regions. We apply robust extreme value analysis methods, including single-gauge and regional frequency approaches, to estimate return levels for 1-, 3-, 6- and 24-hour durations and for 10-, 30-, and 100-year return levels. These are then combined to give IDF curves for each rain gauge, providing an openly accessible, traceable, and reproducible resource for hydrological modelling, engineering design, flood-risk assessment and climate-resilience planning. This dataset represents a step change in accessibility and precision for global IDF estimation and enables a wide range of cross-disciplinary applications.

## Background & Summary

Understanding the dynamics of short-duration intense rainfall is essential for assessing flash flood risks, landslides, and for designing resilient drainage infrastructure^1^. Extreme rainfall is consistently the most dangerous weather event globally in terms of both loss of life and economic damages, including hurricanes and flooding^2,3^. The changing frequency and extent of flooding due to climate change necessitate a deeper understanding of these phenomena^4^. Long-term rainfall records are crucial for studying extremes, particularly sub-daily data, which are difficult to obtain. Return levels are used for various applications and require extensive data records.

Despite advancements in rainfall measurement methods and increased data availability, records are still often short and area-averaged, making them inadequate for capturing extremes. For instance, radar data, available globally since the early 2000s, still lack complete coverage and are impacted by numerous errors, and satellite data, which also have quality concerns, do not accurately represent rainfall on the ground^5^. Rain gauges, in use since the 1800s, provide point estimates; however, are often only available as daily observations for long records, have limited spatial coverage and contain sampling uncertainties^6^. Efforts to create sub-daily datasets and to improve the spatial and temporal resolution of rainfall data^7–9^ have enhanced the coverage and characterisation of sub-daily rainfall, including the need for the development of extensive new quality control methods^10^.

Extreme value analysis (EVA)^11^ is a general statistical framework widely used to estimate the probability of rare events. EVA is used in hydrology to estimate the probability of (often point-based) extreme rainfall events even with limited records, including extreme rainfall. This can be implemented to estimate the frequency of extreme rainfall over various durations and return levels, producing intensity-duration-frequency (IDF) curves crucial for engineering design. Additional methods to augment information using nearby rain gauges with similar frequency distributions using regional frequency analysis (RFA) can also be implemented^12^, improving point return level estimates^13,14^.

Existing parameterisations exist for regional-scale IDF datasets based on satellite observations^15,16^, suggesting the GEV is a good fit. Existing global approaches^17^ rely on gridded reanalysis datasets which systematically underestimate short-duration extremes even after imposing areal reduction factors, especially in the tropics^18^. Whilst attempts have been made to improve grid based IDF curves with point observations^19^ there is currently no global dataset of IDF curves derived directly from quality-controlled sub-daily rain-gauge observations. Point-based gauge records remain fragmented, inconsistently processed and difficult to access but this lack of a harmonised, observationally based global IDF dataset limits robust flood-risk assessment and climate-resilience planning.

We aim to provide a dataset of global IDF curves for a global quality-controlled hourly rain gauge dataset, a new benchmark for global rainfall extremes, offering openly accessible, quality-controlled, sub-daily IDF curves with for real-world decision-making.

The remainder of this paper is organised as follows. The *Methods* section describes the data sources and extraction procedures used to derive annual maxima (AMAX) time-series and outlines the methodology for EVA including single gauge and regional frequency approaches. The structure, content, and accessibility of the GSDR-IDF dataset is detailed in *Data Records* followed by quality control and validation procedures given in the *Technical Validation* section. Potential applications and limitations of the GSDR-IDF are then briefly discussed in the *Usage Notes*.

## Methods

**Fig. 1:**
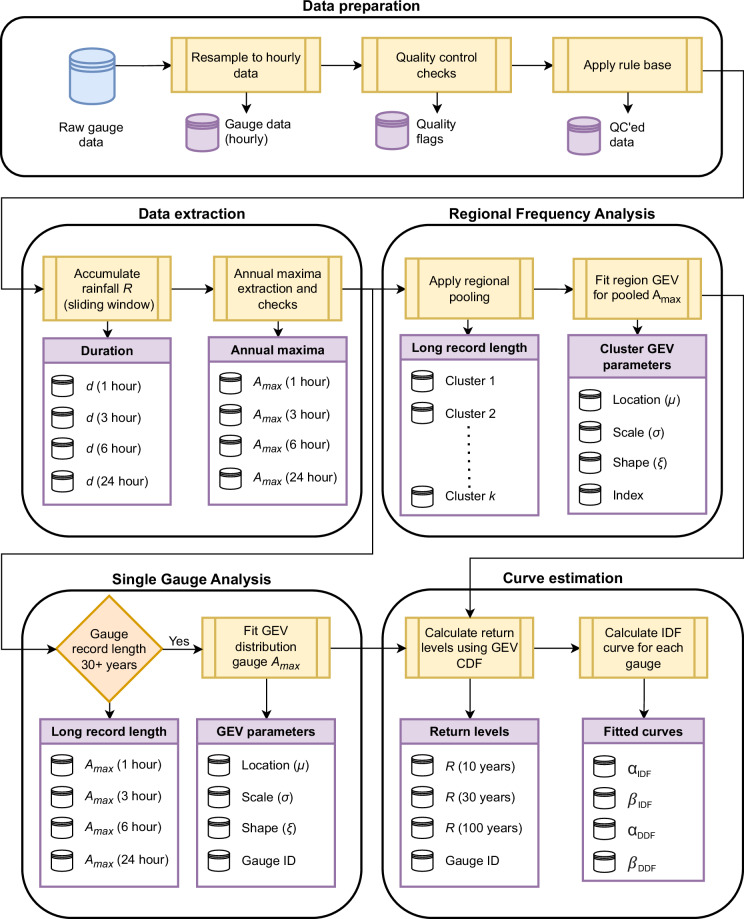
Framework for the methodology for estimating IDF curves for the GSDR-IDF dataset.

This paper represents the culmination of extensive previous work. Figure 1 outlines the main framework for obtaining and presenting this dataset, which is split into four stages, summarised below.

1. **Data preparation**: Synthesis of the data preparation and extraction steps used to obtain AMAX time-series for extreme value analyses and quality control.
2. **Data extraction**: Subsequent pre-processing and selection of rain gauges.
3. **Extreme Value Analysis:** Both Single Gauge Analysis (SGA) and Regional Frequency Analysis (RFA) methodology.
4. **Curve estimation:** Intensity- and Depth-duration frequency curve estimation.

*Data Preparation* was performed in^9^, with subsequent *Data Extraction* including pre-processing, selection of rain gauges and *Extreme Value Analysis* (EVA) performed in^18^. This includes both Single Gauge Analysis (SGA) and Regional Frequency Analysis (RFA), from which the resulting return levels are leveraged to compute Intensity-Duration-Frequency (IDF) curves.

### Data preparation

The Global Sub-Daily Rainfall Dataset (GSDR) provides hourly quality-controlled rain gauge observations^8,9^, which includes 24,394 rain gauges with hourly (or shorter) resolution, the largest and first dataset of global gauge-based hourly rainfall data. Hourly rain gauge data record length (>1 to 104 years), density and quality vary across the globe and so a rule-based quality control (QC) framework was developed and implemented on the dataset^10^.

### Data extraction

Rain gauge data was further pre-processed in^18^ to ensure quality and suitability for EVA^18^ to ensure quality and suitability for EVA. This included four key steps: (1) selecting gauges with sufficient record length and minimal missing data; (2) creating time-series of various durations (3 h, 6 h and 24 h) from 1 h observations; (3) extracting AMAX time-series for those durations; and 4) further selecting gauges based on the completeness and length of their AMAX time-series; <30% of annual maximum values missing and at least 15 (30) years of non-missing annual maxima for RFA (SGA). For the AMAX calculation, a method by^20^ was employed that strategically retains high annual maximum values from years with partial missing data.

### Extreme value analysis

Guerreiro *et al*.^18^ performed both SGA and RFA for gauges across the globe. Both methods were used since some gauges exhibited significantly different return levels, with either SGA or RFA potentially yielding the highest values. Therefore, the simultaneous use of both methods to estimate return levels was recommended for a robust risk assessment in flood infrastructure design. Consequently, this dataset includes estimates from both SGA and RFA where available.

The RFA methodology outlined by Hosking and Wallis^12^ was implemented using the lmomRFA package^21^. This approach involves fitting a Generalized Extreme Value (GEV) distribution to AMAX time-series within homogeneous regions using L-moments. To achieve this at the global level^18^, developed an iterative regionalisation algorithm that first sorts gauges by record length and, for each gauge, iteratively reduces the search radius to identify homogeneous regions. Once a homogeneous region is achieved, its GEV parameters and return levels for 10-, 30- and 100-years are calculated using L-moments, and its gauges are removed from the pool before the process repeats for the remaining gauges.

### Curve estimation

Once the rain gauge GEV parameters were estimated for each combination of method (SGA and RFA) and duration (1, 3, 6 and 24 hours) the return levels for 10-, 30- and 100-years were calculated. These were used to construct DDF curves. A power-law model of the form $D=\alpha {t}^{\beta }$ was then fitted to the depth ($D$) and duration ($t$) data for each return period to capture the scaling relationship. IDF curves were subsequently derived by dividing modelled depths by respective durations, yielding intensity estimates ($I$). The resulting IDF relationships were also expressed using a power-law of the form $I=\alpha {t}^{\beta -1}$. These can then be visualised on log-log axes to highlight the non-linear dependence of rainfall intensity and duration.

## Data Records

As part of the GSDR-IDF dataset, there are multiple stages of the data records, all with different data availability. In this section we outline the data available as part of the GSDR dataset, and the file structure, with a file structure overview given in Table 1.

**Table 1:** A summary of data files and contents as part of the GSDR-IDF dataset.

File name	Description	Contents / Structure
**station-meta.csv**	Station metadata file used for filtering and identifying stations.	Contains station information such as ID, coordinates, record length, start/end timestamps, and data paths.
**station-data.zip**	Compressed folder containing individual station datasets split by method and station.	Files named < *method* > _ < *station*_id > .csv, each containing rainfall frequency analysis results.
**station-curves.zip**	Compressed folder containing intensity–duration–frequency (IDF) curve figures for each station.	Files named < *station_id* > .png
**Filtering-Example.ipynb**	Python notebook file for filtering metadata file to extract relevant station data	Example code snippets for filtering the GSDR-IDF dataset

The file contents of the metadata CSV file (*station-metadata.csv)* is given in Table 2. The information in the meta data file can be used to identify relevant stations (e.g. filter by method, location or country), and gives the station data path for a given method and station (*< method > _ < station_id > .csv*) and IDF curve (*< station > .png)*.

**Table 2:** A description of file contents for the station metadata file used for filtering and identifying stations (station-metadata.csv).

Attribute name	Data type	Description
**Station ID**	string	Unique identifier of station
**Latitude**	float	Station coordinate latitude
**Longitude**	float	Station coordinate longitude
**Country**	string	Country code
**Record length**	float	Record length of station data (years)
**Start**	datetime	Record start timestamp
**End**	datetime	Record end timestamp
**RFA**	string	Path to regional frequency analysis data (blank if none available)
**SGA**	string	Path to single gauge analysis data (blank if none available)
**PXR2.point**	bool	True if comparison to PXR2.point calculated
**Curve**	string	Path to intensity–duration–frequency curve figure (.png)

A breakdown of the file contents for the station CSV files in the compressed data folder *idf-data.zip* is given in Table 3. This includes GEV distribution fitted parameters, 10-, 30- and 100-year return level estimates and record length (years) used to fit the distribution. For RFA only, an additional GEV index parameter is included and a comparison between return level estimates and corresponding values based on PXR2.point for comparison (where applicable). PXR2.point is the corresponding return level estimated in^17^ from the PXR2 dataset (Parametrized eXtreme Rain – 2 parameters), adjusted to get a point equivalent value using areal reduction factors^18^.

**Table 3:** A summary of the subfile contents for each CSV file (< method > _ < station_id > .csv) within the compressed folder idf-data.zip, containing individual station datasets split by analysis method (method) and station ID (station_id).

Column name	Data type	Description
**location**	float	Location parameter of the fitted GEV distribution
**scale**	float	Scale parameter of the fitted GEV distribution
**shape**	float	Shape parameter of the fitted GEV distribution
**index**	string/int	Index flood parameter of the fitted GEV distribution (RFA case only)
**years**	float	Record length (years) used for fitting
**rp10**	float	Rainfall intensity estimate for 10-year return period
**rp30**	float	Rainfall intensity estimate for 30-year return period
**rp100**	float	Rainfall intensity estimate for 100-year return period
**rp10_vs_PXR2**	float (optional)	Comparison or ratio to PXR2 for 10-year event (RFA case only, if available)
**rp30_vs_PXR2**	float (optional)	Comparison or ratio to PXR2 for 30-year event (RFA case only, if available)
**rp100_vs_PXR2**	float (optional)	Comparison or ratio to PXR2 for 100-year event (RFA case only, if available)

**Fig. 2:**
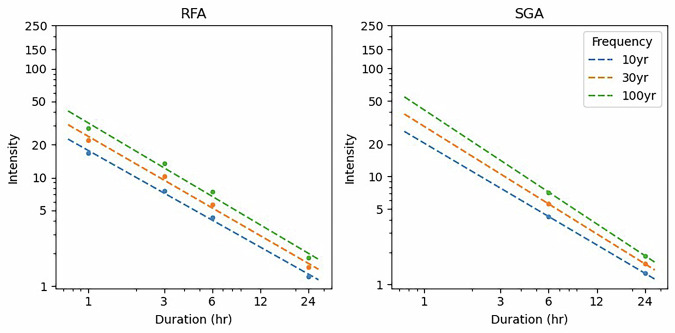
Intensity-duration-frequency curves for RFA (left) and SGA (right) for Station ID 3409 in the United States of America for 10–30- and 100-year return periods.

Figure 2 show an example of an example IDF curve (*US_003409.png*), stored in *idf-figures.zip*, for a station in the United States of America with station ID 3409, where both RFA and SGA estimates are available.

## Technical Validation

To ensure the reliability and reusability of the GSDR-IDF dataset, extensive QC and validation procedures were implemented at each stage of the data pipeline.

### Input data

The underlying hourly rain gauge dataset^8,9^ which forms the GSDR was subjected to a robust rule-based QC framework^10^. This includes automated QC with 25 flags and 11 rules to identify and remove suspicious observations, informed by known errors identified in rain gauge observations. These include comparison checks with daily and monthly data, nearby neighbours and world records, as well as breakpoint and streak checks to identify irregular or intermittent sequences in records.

Annual maxima records for the GSDR are available as part of the GSDR-I climate indices dataset^22,23^. To maximise data availability for AMAX time-series whilst ensuring statistical robustness for extreme value analyses, AMAX values derived from years with over 30% missing observations are retained if their magnitude falls within the top 60% of the AMAX time-series, following the methodology described by^20^. Between 4,065 and 4,181 gauges (17% of the GSDR dataset) have at least 15 years of data (depending on duration) and could therefore be used for RFA. Of these, between 2,018 and 2,053 (8%) had records of 30 or more years, and were therefore available for single gauge analysis.

For SGA, gauges were checked for: autocorrelation (using the Kendall correlation coefficient), trends (using the Mann-Kendall test) and goodness-of-fit of the GEV distribution to the annual maximum time-series (using Kolmogorov-Smirnov Goodness-of-Fit Test). Gauges with significant trends or unsatisfactory GEV fits were excluded. Autocorrelation was present in 70%–84% of annual maximum time-series, and it had no significant impact on the shape parameter distribution (tested using KS test). Consequently, these gauges were retained for the study.

### Extreme value analysis

The Generalised Extreme Value (GEV) distribution is widely supported for modelling short-duration rainfall extremes using a block-maxima approach. Although rare-event estimates are sensitive to the shape parameter, the GEV has been shown to produce realistic short-duration intensities in semi-arid and temperate regions, including Delhi, Brazil, and central Italy, subject to data quality and record length constraints^24–27^. The reliability of GEV fits is further improved through regional pooling and scaling approaches, particularly where high-resolution records are limited^18,28^. While heavier-tailed or non-stationary models may be preferable in very arid regions or under strong climate trends^25,29,30^, these approaches often require longer records and introduce greater uncertainty, limiting their applicability for global analyses^31^. Overall, the GEV provides a robust and pragmatic framework for short-duration extreme rainfall estimation given widespread data constraints.

GEV parameters were estimated using L-moments, a well-established and robust approach for EVA. Previous studies have demonstrated that both SGA and RFA provide consistent and reliable methods for EVA of rainfall AMAX. Where gauge records are sufficiently long, both SGA and RFA are included in the dataset to improve robustness and enable a direct comparison between methods. Accurate estimation of the shape parameter is crucial for robust flood risk assessments, and RFA typically exhibits lower variability in the shape parameter due to regional pooling. GEV fits were found to adequately capture hourly and multi-hour rainfall extremes, with goodness-of-fit tests confirming that the distribution reliably represents short-duration return levels.

For SGA, the fit of the GEV distribution was tested using Kolmogorov-Smirnov Goodness-of-Fit Test. Very few gauges showed a poor GEV fit, and they were not used for SGA. For RFA, clusters were identified for each duration (1–24 hours), and between 233–371 clusters were identified, with an average maximum distance between gauges of 322 km. Each cluster yields GEV parameters (location, scale, shape and index) and cluster IDs. Across the globe, the average distance between gauges for RFA is 1,112 to 1,196 km (varying slightly for different durations due to data availability). This is 20% smaller than for SGA, with large areas of sparse gauge coverage, particularly in Africa and South America. This is in contrast with the high station densities in Europe and the USA. For SGA, up to 56% of gauge records are not sufficiently long, highlighting the necessity for RFA.

Candidate regions, defined by distance, are evaluated using L-moment ratios to test for gauge discordance, homogeneity, and GEV goodness-of-fit^12^. Discordance and homogeneity are assessed using the discordance measure and the heterogeneity measure defined by Hosking and Wallis^12^, with the latter involving a comparison of observed L-moment ratios against simulated homogeneous regions generated via Monte Carlo sampling. Finally, the quality of the regional GEV goodness-of-fit was assessed by comparing its L-kurtosis to the regional average L-kurtosis using standard deviations derived from the Monte Carlo simulations. RFA regions (>4 gauges) are created when the candidate region shows no discordant gauges, is not heterogeneous and the regional GEV fit is acceptable at the 10% significance level.

Regional GEV parameters and return levels (10-, 30-, and 100-year) are then estimated using L-moments. A comparison was performed between SGA/RFA return level estimates and the corresponding grid cell estimates obtained from the PXR2 dataset^17^, based on ERA5 reanalysis data. As this is a gridded dataset (~961 km² per grid cell), modified return level estimates – PXR2.point – were derived to allow for better comparison with the point gauge data in^18^. This was done by dividing PXR2 return levels by areal reduction factors (ARFs). These ARFs are given by 0.64, 0.76, 0.82 and 0.89 for durations 1-, 3-, 6- and 24-hour durations, given in^32^. They correct for spatial averaging inherent in gridded data, aiming to approximate what the return levels would be at a single point.

Comparisons between datasets and methods were made for gauges or grid cells where results from all 3 methods (1801–1842 gauges) were available (SGA, RFA and PXR2.point). From these records, the IDF curves have been calculated for gauges, giving 23,985 fitted curves for return levels of 10-, 30- and 100-years.

### Spatial consistency

While RFA incorporates additional data, effectively doubling the number of gauges globally where EVA can be applied, its clustering approach can distort return levels. Specifically, when an individual gauge captures an extreme event not observed by others in the same cluster, RFA will reduce that gauge’s return levels compared to SGA, while simultaneously increasing the return levels of other gauges within the cluster. This may benefit the other gauges but disadvantage the gauge that recorded the extreme event^18^. Return levels and GEV parameters are visually and statistically compared across neighbouring gauges.

**Fig. 3:**
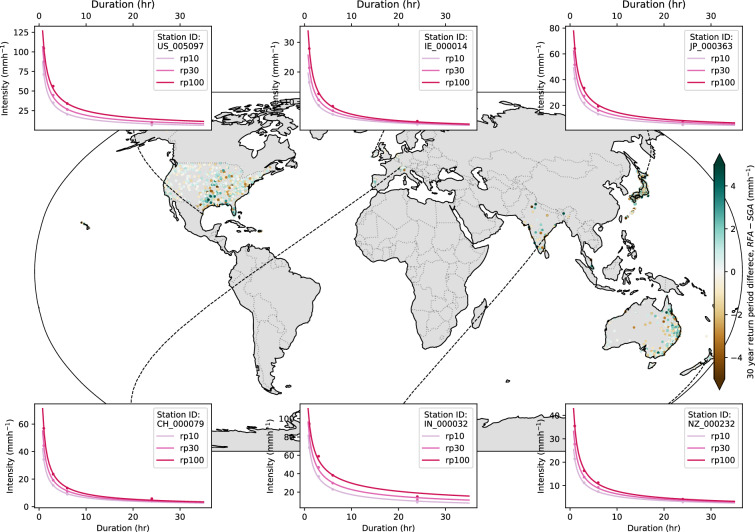
The difference between 30-year return periods for RFA and SGA (RFA – SGA) for all stations with RFA estimates, with example intensity-duration-frequency (IDF) curves for a station in the United States (US), Ireland (IE), Japan (JP), Switzerland (CH), India (IN) and New Zealand (NZ), respectively, using RFA to estimate return periods for 10, 30 and 100 years.

Figure 3 shows the IDF curves for 6 example stations in countries with dense gauge networks, where return levels have been obtained using RFA, overlayed on a map of the absolute difference between the 30-year return period for RFA and SGA. Regional differences between return levels are evident but are generally small, and while median differences are limited across regions, notable absolute differences can occur at individual sites depending on local versus regional extreme rainfall behaviour. The magnitude and spatial pattern of differences between RFA and SGA return levels vary by location and duration, reflecting heterogeneity in gauge density and rainfall behaviour. This variability and its implications for regional versus gauge-level estimates have been discussed in detail in^18^.

**Fig. 4:**
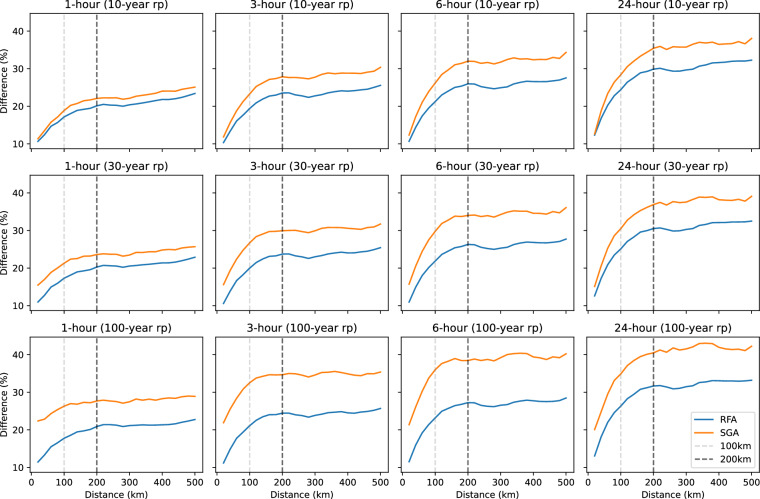
Percentage difference in return level estimates between rain gauge pairs as a function of inter-gauge separation distance for 10-, 30- and 100-year return periods (rp) and durations of 1, 3, 6 and 24 hours. Only gauge pairs separated by less than 500 km are included. Vertical lines indicate 100 km and 200 km distance thresholds.

To assess how spatial separation between rain gauges influences the representativeness of sub-daily rainfall extremes, differences in return level estimates between neighbouring gauges were evaluated as a function of inter-gauge distance. Figure 4 shows the percentage difference (relative difference) in return level estimates between gauge pairs for 10-, 30- and 100-year events across durations of 1, 3, 6 and 24 hours, using all gauge pairs separated by less than 500 km. The 100 km and 200 km distance thresholds adopted in this dataset are shown to highlight distances beyond which inter-gauge differences increase and spatial representativeness declines. The variability in return level differences is lower for both shorter return periods and smaller separation distances. For all durations and return periods, there is a significant increase in the difference between gauge estimates as the separation distance increase up to 200 km, beyond which the difference appears to stabilise. Furthermore, SGA consistently exhibits greater variability in return level differences than RFA and the absolute difference in return levels becomes smaller for longer durations.

For a duration of 1 hour using SGA, the average difference between 10-year return levels for gauges with separation distances 1–100 km and 100–200 km are 16.5% and 21.2% respectively, while it becomes 54% past 300 km. Differences are larger for a duration of 24 hours, with average differences of 24.5% (1–100 km) and 33.2% (100–200 km). Given that IDF curves require all available durations for fitting, a global distance threshold of 100 km is proposed. Extrapolating IDF curves for distances beyond 100 km should be considered only with caution.

## Usage Notes

The GSDR-IDF dataset provides globally consistent IDF curves derived from quality controlled hourly rain gauge observations. When applying this dataset users should consider the following practical guidance and limitations.

### Temporal coverage and stationarity

The GSDR-IDF is a static dataset, with the most recent AMAX data extending to 2019, and many records ending before 2015. While updates to the GSDR dataset are in progress, they are not yet integrated into the current version of GSDR-IDF. As such, the derived curves represent historical climatic conditions and do not incorporate recent changes in rainfall extremes due to climate change or any potential non-stationarity within records. Users applying the data for contemporary design or adaptation purposes should consider whether adjustments or non-stationary analyses are required.

### Spatial representativeness and distance thresholds

All IDF curves represent point-based estimates, and although regional frequency analysis was implemented, the generalisability of estimated parameters to ungauged locations is not recommended. Sub-daily rainfall exhibits substantial spatial variability, particularly at 1- and 3-hour durations, so curves may not reflect conditions beyond the immediate vicinity of a gauge. For ungauged locations accessed via the data portal, curves are displayed only for stations within a maximum distance of 200 km, with a cautionary warning for 100–200 km. This threshold is derived from cross-gauge analysis of return level differences (see Technical Validation).

Users should avoid applying IDF curves beyond 200 km, as representativeness declines rapidly, and it is also recommended that users also ensure that analyses exclude oceanic or non-land grid points when mapping or interpolating IDF curves, as rainfall climatology differs markedly between marine and terrestrial environments.

### Extrapolating between different fitted duration ranges

The IDF curves are constructed using AMAX in the range 1–24 hours, therefore extrapolating to durations outside this range is not recommended, as the power law relationship is not validated outside this domain.

### Modelling uncertainty

Each station within the GSDR-IDF dataset may include a return level estimate from SGA and RFA, each which have their own strengths. SGA preserves the unique extreme behaviour of an individual gauge but can be highly uncertain when record lengths are short. RFA reduces parameter uncertainty through regional pooling but may slightly dampen unusually extreme events recorded at a single gauge if neighbouring stations do not exhibit similar behaviour. Uncertainty increases with return period. Where gauge records are short, the 100-year return levels should be interpreted with caution, as sampling variability remains substantial despite L-moment fitting. Users undertaking risk-averse design may prefer to consider the upper bound across methods.

### Data sparse regions

Large areas across the world have limited sub-daily records, particularly records with sufficient data length to robustly apply SGA. In these regions, there may be estimates using RFA, however clusters may span larger geographic extents increasing regional heterogeneity. As short-duration rainfall extremes (e.g. 1-hour) are challenging to estimate in these cases, users working in data-sparse areas should consider complementing GSDR-IDF with additional local data sources (e.g., radar or satellite products) or broader uncertainty assessments.

### Data portal and reproducibility

The GSDR-IDF Data Portal (https://amyycb.shinyapps.io/gsdr-idf) provides interactive access to station metadata, GEV parameters, RFA clusters, return levels, and IDF curves. Users can query locations by map or address and download figures (.png) and station-level return level files (.csv). All underlying code is openly available on GitHub (https://github.com/amyycb/globalextremes), supporting reproducibility and enabling users to integrate the dataset into customised workflows. The portal supports applications in hydrology, engineering, and water resource management by providing quality-controlled IDF curves for sub-daily rainfall extremes.

## Data Availability

The dataset described in this study is publicly available as part of the GSDR-IDF dataset archived on 10.5281/zenodo.18152624^33^. The data are provided in both.csv and.png format, with comprehensive metadata and example code for filtering also included.

## Code availability

The code used in this study is publicly available as part of the GSDR-IDF dataset archived on Zenodo: Green, A. C. (2026). GSDR-IDF: Global Intensity-Duration-Frequency curves based on observed sub-daily rainfall [Data set]. Zenodo. 10.5281/zenodo.18152624^33^

## References

[1] Fowler, H. *et al*. Towards advancing scientific knowledge of climate change impacts on short-duration rainfall extremes. *Phil. Trans. R. Soc. A* **379**, 2195, 10.1098/rsta.2019.0542 (2021). 10.1098/rsta.2019.0542 33641464

[2] Fowler, H. J. *et al*. Precipitation extremes in 2023. *Nat. Rev. Earth Environ.* **5**, 250–252, 10.1038/s43017-024-00547-9 (2024).

[3] Green, A. C. *et al*. Precipitation extremes in 2024. *Nat. Rev. Earth Environ.* **6**, 243–245, 10.1038/s43017-025-00666-x (2025).

[4] Hettiarachchi, S., Wasko, C. & Sharma, A. Increase in flood risk resulting from climate change in a developed urban watershed – the role of storm temporal patterns. *Hydrol. Earth Syst. Sci.* **22**, 2041–2056, 10.5194/hess-22-2041-2018 (2018).

[5] Tapiador, F. *et al*. Global precipitation measurements for validating climate models. *Atmos. Res.* **197**, 1–20, 10.1016/j.atmosres.2017.06.021 (2017).

[6] Dunn, R. E., Fowler, H. J., Green, A. C. & Lewis, E. Tipping-bucket rain gauges: a review of the undercatch phenomenon, and methods for its reduction and correction. *Weather.* **80**, 196–205, 10.1002/wea.7736 (2025).

[7] Blenkinsop, S. *et al*. The INTENSE project: using observations and models to understand the past, present and future of sub-daily rainfall extremes. *Adv. Sci. Res.* **15**, 117–126, 10.5194/asr-15-117-2018 (2018).

[8] Lewis, E. *et al*. GSDR: A Global Sub-Daily Rainfall Dataset. *J. Clim.* **32**, 4715–4729, 10.1175/JCLI-D-18-0143.1 (2019).

[9] Lewis, E. *et al*. Subset of Global Sub-Daily Rainfall (GSDR) dataset [Data set]. *Zenodo*10.5281/zenodo.8369987 (2019).

[10] Lewis, E. *et al*. Quality control of a global hourly rainfall dataset. *Environ. Model. Softw.* **144**, 105169, 10.1016/j.envsoft.2021.105169 (2021).

[11] Coles, S., Bawa, J., Trenner, L. & Dorazio, P. *An Introduction to Statistical Modeling of Extreme Values*, 2nd ed. Springer, London, UK (2001).

[12] Hosking, J.R.M. & Wallis, J.R. *Regional Frequency Analysis*, IBM T J Watson Research Center, New York, James R. Wallis (1997).

[13] Liang, Y. *et al*. L-Moment-Based Regional Frequency Analysis of Annual Extreme Precipitation and its Uncertainty Analysis. *Water Resour. Manage.* **31**, 3899–3919, 10.1007/s11269-017-1715-5 (2017).

[14] Debbarma, N. *et al*. Uncertainty Analysis of Regional Rainfall Frequency Estimates in Northeast India. *Civil Eng. J.* **7**(11), 1817–1835, 10.28991/cej-2021-03091762 (2021).

[15] Noor, M. *et al*. Evaluating intensity-duration-frequency (IDF) curves of satellite-based precipitation datasets in Peninsular Malaysia. *Atmos. Res.* **248**, 105203, 10.1016/j.atmosres.2020.105203 (2021).

[16] Zeri, S. J., Hamed, M. M., Wang, X. & Shahid, S. Utilizing satellite data to establish rainfall intensity-duration-frequency curves for major cities in Iraq. *Water* **15**, 852, 10.3390/w15050852 (2023).

[17] Courty, L. *et al*. Intensity-duration-frequency curves at the global scale. *Environ. Res. Lett.* **14**, 084045, 10.1088/1748-9326/ab370a (2018).

[18] Guerreiro, S. B. *et al*. Unravelling the complex interplay between daily and sub-daily rainfall extremes in different climates. *Weather Clim. Extremes* **46**, 100735, 10.1016/j.wace.2024.100735 (2024).

[19] Lau, A. & Behrangi, A. Understanding Intensity–Duration–Frequency (IDF) Curves Using IMERG Sub-Hourly Precipitation against Dense Gauge Networks. *Remote Sens.* **14**, 5032, 10.3390/rs14195032 (2022).

[20] Papalexiou, S. M. & Koutsoyiannis, D. Battle of extreme value distributions: A global survey on extreme daily rainfall. *Water Resour. Res.* **49**, 187–201, 10.1002/wrcr.20073 (2013).

[21] Hosking, J.R.M. & Wallis, J.R. Regional frequency analysis using L-moments. In *Hydraulic Engineering: Saving a Threatened Resource—In Search of Solutions*, 13–18. ASCE, Baltimore, Maryland (2019).

[22] Pritchard, D. *et al*. An Observation-Based Dataset of Global Sub-Daily Precipitation Indices (GSDR-I). *Sci. Data* **10**, 393, 10.1038/s41597-023-02238-4 (2023). 37349333 10.1038/s41597-023-02238-4 PMC10287747

[23] Pritchard, D. *et al*. GSDR-I Global Sub-Daily Precipitation Indices - Dataset (0.1.0) [Data set]. *Zenodo*10.5281/zenodo.7492812 (2022).

[24] Chaudhuri, R. & Sharma, P. Addressing uncertainty in extreme rainfall intensity for semi-arid urban regions: case study of Delhi, India. *Nat. Hazards* **104**, 2307–2324, 10.1007/s11069-020-04273-5 (2020).

[25] Pansera, W. & Gomes, B. Performance of multiparameter distributions in estimating rainfall extremes and deriving IDF equations in Paraná. *Rev. Bras. Recur. Hídricos* **30**(3), 2318–0331, 10.1590/2318-0331.302520240060 (2025).

[26] Ballarin, A. *et al*. Combined predictive and descriptive tests for extreme rainfall probability distribution selection. *Hydrol. Sci. J.* **67**, 1130–1140, 10.1080/02626667.2022.2063725 (2022).

[27] Gentilucci, M. *et al*. GEV Analysis of Extreme Rainfall: Comparing Different Time Intervals to Analyse Model Response in Terms of Return Levels in the Study Area of Central Italy. *Sustainability* **15**(15), 11656, 10.3390/su151511656 (2023).

[28] Blanchet, J. *et al*. A regional GEV scale-invariant framework for Intensity-Duration-Frequency analysis. *J. Hydrol.* **540**, 82–95, 10.1016/j.jhydrol.2016.06.007 (2016).

[29] Kyojo, E. *et al*. Modeling non-stationarity in extreme rainfall data and implications for climate adaptation: A case study from southern highlands region of Tanzania. *Sci. Afr.* **25**, 2321, 10.1016/j.sciaf.2024.e02321 (2024).

[30] Ghalavand, M. *et al*. Modeling Return Levels of Non-Stationary Rainfall Extremes Due to Climate Change. *Atmosphere* **16**(2), 136, 10.3390/atmos16020136 (2025).

[31] Hossain, I. *et al*. Comparison of estimation techniques for generalised extreme value (GEV) distribution parameters: a case study with Tasmanian rainfall. *Int. J. Environ. Sci. Technol.* **19**, 7737–7750, 10.1007/s13762-021-03693-5 (2021).

[32] Kjeldsen, T. R. *et al*. Revitalisation of the FSR/FEH rainfall-runoff method. Final Report to DEFRA/EA. Wallingford (2005).

[33] Green, A. C. *et al*. GSDR-IDF: Global Intensity-Duration-Frequency curves based on observed sub-daily rainfall. *Zenodo*10.5281/zenodo.18152624 (2026). 10.1038/s41597-026-06858-4 PMC13018227 41690990

